# Etiological and epidemiological characteristics of surgically treated radial nerve lesions: A 20-year single-center experience

**DOI:** 10.3389/fsurg.2022.942755

**Published:** 2022-09-20

**Authors:** Lukas Rasulić, Slavko Đjurašković, Novak Lakićević, Milan Lepić, Andrija Savić, Jovan Grujić, Aleksa Mićić, Stefan Radojević, María Elena Córdoba-Mosqueda, Jacopo Visani, Vladimir Puzović, Vojin Kovačević, Filip Vitošević, Stefan Mandić-Rajčević, Saša Knezevic

**Affiliations:** ^1^ Faculty of Medicine, University of Belgrade, Belgrade, Serbia; ^2^ Department of Peripheral Nerve Surgery, Functional Neurosurgery and Pain Management Surgery, Clinic for Neurosurgery, University Clinical Center of Serbia, Belgrade, Serbia; ^3^ Clinic for Neurosurgery, Clinical Center of Montenegro, Podgorica, Montenegro; ^4^ Clinic for Neurosurgery, Military Medical Academy, Belgrade, Serbia; ^5^ Department of Neurology and Neurosurgery, Hospital Central Sur de Alta Especialidad PEMEX, Mexico, Mexico; ^6^ Department of Neurosurgery, Santa Maria Della Misericordia Hospital, Rovigo, Italy; ^7^ “College of Sport and Health, Belgrade, Serbia; ^8^ Faculty of Medical Sciences, University of Kragujevac, Kragujevac, Serbia; ^9^ Clinic for Neurosurgery, Clinical Center of Kragujevac, Kragujevac, Serbia; ^10^ Interventional Neuroradiology Department, Center for Radiology and MRI, Clinic for Neurosurgery, University Clinical Center of Serbia, Belgrade, Serbia; ^11^ School of Public Health and Health Management and Institute of Social Medicine, Faculty of Medicine, University of Belgrade, Belgrade, Serbia; ^12^ Center for Anesthesiology, Resuscitation and Pain Therapy, University Clinical Centre of Serbia, Belgrade, Serbia

**Keywords:** radial nerve, etiology, epidemiology, mechanism of injury, surgery

## Abstract

**Introduction:**

Radial nerve lesions present a clinical entity that may lead to disability, psychological distress, and job loss, and thus requires great attention. Knowledge of the etiology and exact mechanism of the nerve impairment is of great importance for appropriate management of these patients, and there are only a few papers that focused on these features in patients with surgically treated radial nerve lesions. The lack of studies presenting the etiology and injury mechanisms of surgically treated radial nerve lesions may be due to a relatively small number of specialized referral centers, dispersion to low-flow centers, and a greater focus on the surgical treatment outcomes.

**Aim:**

The aim of this study was to describe the etiological and epidemiological characteristics of patients with surgically treated radial nerve lesions of various origins.

**Methods:**

This retrospective study evaluated 147 consecutive patients with radial nerve lesion, treated in the department during the last 20 years, from January 1, 2001, until December 31, 2020.

**Results:**

The majority of patients belonged to the working population, and 70.1% of them were male. Most commonly, the etiology of nerve lesion was trauma (63.3%) or iatrogenic injury (28.6%), while the less common origin was idiopathic (4.1%) or neoplastic (4.1%). The most frequent location of the lesion was in the upper arm, followed by the elbow and forearm. Fracture-related contusion was the most common mechanism (29.9%), followed by postoperative fibrosis (17.7%), lacerations (17.7%), and compression (15.6%).

**Conclusion:**

Based on the fact that traumatic or iatrogenic injuries constitute the majority of cases, with their relevant mechanisms and upper arm predomination, it is crucial to raise awareness and understanding of the radial nerve injuries among orthopedic surgeons to decrease the numbers of these patients and properly preserve or treat them within the initial surgery.

## Introduction

Radial nerve lesions present a clinical entity that may lead to functional loss (1), disability (2), psychological distress (3), and job loss (4) and should be, therefore, recognized as a significant socioeconomic problem (5, 6). Knowledge of the etiology and exact mechanism of the nerve impairment is of great importance for appropriate management of these patients (7), and there are only a few papers that focused on these features in patients with surgically treated radial nerve lesions (8, 9).

While many of the posture or compression-related radial nerve palsies may recover spontaneously, as do some of the contusion lesions associated with bone fractures (10, 11), radial nerve lesions demanding surgery are most commonly caused by trauma (8, 12), unlike lesions of the median and ulnar nerve, whose origin is most often idiopathic entrapment (13). While the frequency of iatrogenic radial nerve lesions referred for surgery is similar to that of other major nerves of the arm (14), neoplastic lesions are rare and account only for a small portion of all peripheral nerve tumors (15).

The radial is the deep seated nerve, adjacent to the bones and frequently subjected to fracture-related contusion (8, 16) or laceration (16–18), by the rule a consequence of humeral shaft fracture in the upper arm (8, 19), while the lesions of the trunk or its main branches in the distal parts of the arm are mostly associated with the elbow, radius, and ulna fractures (8). Less frequently, the nerve may be compressed, contused, lacerated, or cut without an associated bone fracture (8, 9, 20–23).

Because of its frequent association with humeral shaft fracture (24), the majority of studies concerning radial nerve lesions have focused on patients with this associated fracture (10, 16–18, 20, 25). The lack of studies presenting the etiology and injury mechanisms of surgically treated radial nerve lesions may also be due to a relatively small number of specialized referral centers, dispersion to low-flow centers, and a greater focus on the surgical treatment (26).

The aim of this study was to describe the etiological and epidemiological characteristics of patients with surgically treated radial nerve lesions of various origins in a single-center during a 20-year period.

## Materials and methods

### Patients

This is a retrospective study that included 147 consecutive patients with radial nerve lesion treated at the Department for Peripheral Nerve Surgery, Functional Neurosurgery and Pain Management Surgery, Clinic for Neurosurgery, University Clinical Center of Serbia, in Belgrade, Serbia, in a 20-year period from January 1, 2001, to December 31, 2020.

The patients with radial nerve lesions were included in the study according to the following criteria:

#### Inclusion criteria

• Patients with ultrasonography and electromyoneurography verified radial nerve lesion referred for surgery and treated during the study period.
• Radial nerve lesion located in the upper arm, elbow, or forearm region.
• Lesion of the radial nerve main branches (deep-motor and superficial-sensitive).
• Posterior interosseous nerve (PIN) lesion.
• Superficial sensory radial nerve (SSRN) lesion.

#### Exclusion criteria

• Patients with radial nerve lesion undergoing conservative treatment.
• Radial nerve lesion in the infraclavicular region, as the part of brachial plexus injury.

### Data retrieval

All data in the study were obtained by reviewing patients’ hospital records and follow-up examinations. We collected data on age (<25, 26–50, 51–75), gender (male/female), whether belonging to the working-age population (27), area of residence (urban/rural), tobacco smoking (yes/no), associated diseases, etiology of nerve lesion (traumatic/iatrogenic/neoplastic/idiopathic), and mechanism of nerve injury. In addition, for patients with traumatic injuries, we noted the energy of the trauma (high-energy/low-energy), associated injuries, and nerve continuity (preserved/disrupted).

### Statistical analysis

All statistical procedures were performed with SPSS v26.0 software package (IBM Corporation, Armonk, NY, USA). For descriptions of the parameters of interest, we used the methods of descriptive statistics: mean, median, range, absolute (N), and relative (%) frequencies. The normality of data was assessed using the Shapiro–Wilk test. The association between patients’ groups was analyzed using the Chi-square test with a 95% confidence interval, and statistical significance set at *p* < 0.05.

## Results

Out of all studied patients, 104 (70.7%) were male and 43 (29.3%) were female. The patients' age ranged from 12 to 75 years, and the mean age of the population was 38.2 ± 15.3. Two-thirds of the male patients −69 (66.3%) were younger than 40 years (mean age = 35.4), while female patients had more even distribution, counting 24 (55.8%) older than 40 (mean age = 45.1). All patients aged under 18 years were males (12, 14, and 16 years old). The youngest female patient was aged 18, while the oldest male and female patients were aged 72 and 75, respectively.

The majority of studied patients—137 (93.20%)—belonged to the working-age population, and there was a statistically significant difference in the male to female ratio regarding the analyzed age groups: the majority of male patients belonged to the group aged 0–25 years (87.1%), while the most of the women were aged 26–50 years (75.6%). Slightly more than a half of the patients—79 (53.7%)—lived in urban places, while 68 (46.3%) lived in rural places. Comorbidities were present in 43 (29.2%), and 61 (41.5%) patients were tobacco smokers before and at the time of surgery (Table 1).

**Table 1:** Patient distribution with reference to comorbidities and tobacco smoking within gender and age groups.

Comorbidities	Gender		Age groups			Total
	Male	Female	0–25	26–50	51–75	
Chronic hypertension	10	9	—	8	11	19
Diabetes mellitus	4	4	1	4	3	8
Hypothyroidism	—	5	—	4	1	5
Ischemic heart disease	2	—	—	—	2	2
Chronic hypertension and diabetes mellitus	7	2	—	2	7	9
Total	23	20	1	18	24	43
Tobacco smoking	39	22	15	26	20	61

**Figure 1:**
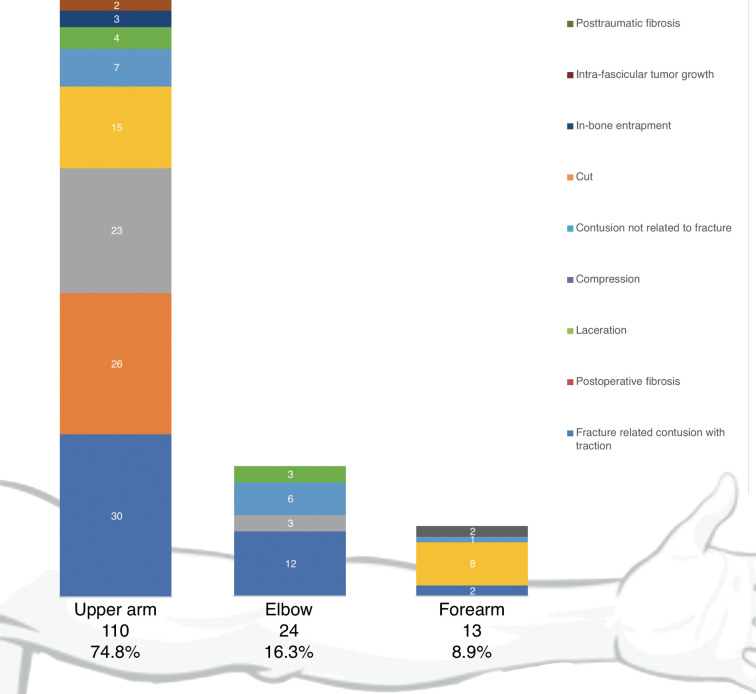
Distribution of the patients with reference to location and mechanism of nerve injury.

The most common location of the nerve lesion was in the upper arm −110 (74.8%), followed by the elbow −24 (16.3%) and forearm −13 (8.8%). Almost all elbow injuries—21 (87.5%)—involved the radial nerve trunk, while only 2 involved both radial nerve main branches. The majority of forearm nerve lesions—11 (84.6%)—involved PIN, while only 2 (15.4%) involved SSRN. The mechanisms of nerve injury at different locations in the upper extremity are presented in Figure 1.

Out of all studied patients, 100 (68.0%) had preserved, while 47 (32.0%) had disrupted nerve continuity (complete vs. partial disruption = 46:1).

Out of the total 147 patients, the majority (129) were trauma patients. Nerve injury in these occurred due to the trauma in 93 (72.1%) patients, while 36 (24.5%) developed iatrogenic nerve injury. The remaining six iatrogenic injuries occurred in nontraumatized patients. Neoplastic and idiopathic nerve lesions involved six patients each (Table 2).

**Table 2:** Distribution of the patients with reference to etiology, among gender, age groups, and location of the nerve lesion.

Etiopathogenesis of nerve lesion	Gender	Age groups			Location of nerve lesion			Total (*n* = 147)
		0–25	26–50	51–75	Upper arm	Elbow	Forearm	
Traumatic	M	24	39	11	51	20	3	93
	f	4	9	6	13	4	2	
Iatrogenic	m	3	11	7	21	—	—	42
	f	2	7	12	21	—	—	
Neoplastic	M	—	4	—	2	—	2	6
	F	—	2	—	2	—	—	
Idiopathic	M	—	4	—	—	—	4	6
	F	—	2	—	—	—	2	

Most of the studied patients—129 (87.7%)—developed nerve lesion due to trauma (high-energy vs. low-energy trauma = 71:58). Males were more commonly injured during road traffic accidents [31 (77.5%)], occupational accidents [27 (87.1%)], and physical confrontation [8 (100%)], while more than a half of the females [20 (54.0%)] were injured during fall from the standing position. Table 3 presents further details on the cause of trauma.

**Table 3:** Cause of trauma, age, and gender distribution in 129 traumatized patients.

Cause of trauma (*n* of patients = 129)	Gender		Age groups			Total
	Male	Female	0–25	26–50	51–75	
Road traffic accident	31	9	13	26	1	40
Fall from the standing position	18	20	8	9	21	38
Occupational accident^a^	27	4	5	13	13	31
Bad posture during sleep	5	3	—	8	—	8
Physical confrontation	8	—	5	3	—	8
Heavy object crushing	2	—	2	—	—	2
Shooting with firearms	1	—	1	—	—	1
Traction by a dog leash	—	1	—	—	1	1
Total	92	37	33	60	36	129

^a^ Occupational accidents included crushing and/or traction by a heavy machine, falls from the height, heavy object crushing, and injuries by a sharp object.

Excluding the radial nerve injury, most of the traumatized patients [110 (85.3%)] had other associated injuries (Table 4), the majority of which [79 (71.9%)] had a humeral shaft fracture.

**Table 4:** Location of nerve lesion and other associated injuries.

Number of other associated injuries (*n* of patients = 110)	Location of nerve lesion			Total
	Upper arm	Elbow	Forearm	
One other associated injury	52	11	1	64
HSF	51	—	—	51
Lateral epicondyle fracture	—	2	—	2
Elbow fracture	—	8	—	8
Ulna fracture	—	—	1	1
Biceps muscle	1	—	—	1
Brachioradial muscle	—	1	—	1
Two other associated injuries	14	9	3	26
Radius and ulna fracture	—	7	2	9
HSF and radius fracture	4	—	—	4
HSF and EJL	2	—	—	2
HSF and HJL	2	—	—	2
HSF and costa (I-II) fracture	1	—	—	1
HSF and ulnar nerve	1	—	—	1
HSF and subscapular muscle	1	—	—	1
Ulna fracture and epidural hematoma	—	—	1	1
Biceps and triceps muscle	3	—	—	3
Biceps and brachioradial muscle	—	1	—	1
Biceps tendon and brachioradial muscle	—	1	—	1
Three other associated injuries	11	—	—	11
HSF, radius, and ulna fracture	4	—	—	4
HSF, HJL, and ulnar nerve	2	—	—	2
HSF, EJL, and costa (I–III) fracture	1	—	—	1
HSF, costa (III–X), and vertebra (T8) fracture	1	—	—	1
HSF, spleen, and mesentery rupture	1	—	—	1
Femur, pelvis bones, and costa (V–III) fracture	1	—	—	1
Femur, pelvis bones, and tibia fracture	1	—	—	1
Four other associated injuries	5	1	—	6
HSF, EJL, radius, and ulna fracture	1	—	—	1
HSF, brachial plexus lesion, radius, and ulna fracture	1	—	—	1
HSF, costa (II–V), vertebra (C2, C3), and femur	1	—	—	1
HSF, vertebra (C2, C3), clavicula, and deltoideus	1	—	—	1
HSF, ulna, femur, and tibia fracture	1	—	—	1
Ulnar and radial artery, ulnar, and median nerve	—	1	—	1
Five other associated injuries	3	—	—	3
HSF, brachial artery, median and ulnar nerve, and hemothorax	1	—	—	1
HSF, median nerve, femur, and fibula fracture	1	—	—	1
HSF, ulnar nerve, ulna, femur, and fibula fracture	1	—	—	1
Total	85	21	4	110

HSF, humeral shaft fracture; EJL, elbow joint luxation; HJL, humeral joint luxation.

Table 5 reviews the causes and mechanisms of traumatic nerve injuries. The most common cause were road traffic accidents −27 (29.0%), occupational accidents −26 (28.0%) and falls from the standing position −20 (21.5%). The most common mechanisms of nerve injury were fracture related contusion −44 (47.3%) and laceration −18 (19.3%). The majority of fracture related contusions −30 (68.2%) were a consequence of humeral shaft fracture, as well as 13 (72.2%) lacerations, and 2 (28.6%) cuts. The elbow fractures resulted in 7 (15.9%) contusions and 1 laceration, while radius and/or ulna fractures resulted in 7 (15.9%) contusions and 2 lacerations. The 14 contusions, 5 cuts, and 2 lacerations, without an associated fracture, were a consequence blunt trauma or injury by a sharp object. Two injuries by a sharp object resulted in posttraumatic fibrosis, while all compression injuries occurred due to bad posture during sleep (Saturday night palsy).

**Table 5:** Causes and mechanisms of traumatic radial nerve injuries.

*N* of patients (=93)	Traumatic nerve lesions	Gender		Age groups			Total
		Male	Female	0–25	26–50	51–75	
Cause of injury	Road traffic accident	22	5	12	15	—	27
	Occupational accident	22	4	2	13	11	26
	Fall from the standing position	14	6	6	9	5	20
	Compression due to bad posture during sleep	5	3	—	8	—	8
	Physical confrontation	8	—	5	3	—	8
	Heavy object crushing as a nonoccupational accident	2	—	2	—	—	2
	Gunshot wound	1	—	1	—	—	1
	Traction by a dog leash	—	1	—	—	1	1
Mechanism of injury	Fracture-related contusion with traction	34	10	11	22	11	44
	Laceration	16	2	7	10	1	18
	Contusion not related to fracture	12	2	6	8	—	14
	Compression	5	3	—	8	—	8
	Cut	7	—	4	—	3	7
	Posttraumatic fibrosis	—	2	—	—	2	2

Table 6 reviews the causes and mechanisms of iatrogenic nerve injuries. The most common cause was open reduction and internal fixation (ORIF) of the humeral shaft [29 (69.0%)]. The most common mechanism of nerve injury associated with ORIF was postoperative fibrosis [20 (69.0%)], while the less common were nerve entrapment between the bone fragments [3 (10.3%)], nerve compression by a plate [3 (10.3%)], and nerve laceration [3 (10.3%)]. The osteosynthesis material removal led to nerve laceration in five patients. All cases of tumor resection (six) resulted in postoperative fibrosis. In two cases, repositioning under general anesthesia led to the compression injury.

**Table 6:** Causes and mechanisms of iatrogenic radial nerve injuries.

*N* of patients (=42)	Iatrogenic nerve lesions	Gender		Age groups			Total
		Male	Female	0–25	26–50	51–75	
Cause of injury	Internal fixation of the humeral shaft	16	13	5	9	15	29
	Osteosynthetic material removal	2	3	—	1	4	5
	Schwannoma resection	1	3	—	4	—	4
	Lipoma resection	2	—	—	2	—	2
	Repositioning under general Anesthesia	—	2	—	2	—	2
Mechanism of injury	Postoperative fibrosis	13	13	2	11	13	26
	Laceration	5	3	1	4	3	8
	Compression	1	4	—	3	2	5
	In-bone entrapment	2	1	2	—	1	3

All patients with neoplastic etiology of the nerve lesion (male vs. female = 4:2) had benign peripheral nerve sheath tumor (PNST), out of whom three had schwannoma (arising from the sensory fibers), two had neurofibroma (arising from the motor fibers), and one had a hybrid tumor with neurofibroma capsule and schwannoma tissue (arising from the sensory fibers). All but two schwannomas in the forearm region originating from the SSRN were located in the upper arm region.

All patients with idiopathic etiology of the nerve lesion (male vs. female −4:2) had PIN entrapment syndrome due to the nerve compression at the supinator muscle arch—the arcade of Frohse.

## Discussion

For more than 40 years, our department is dedicated to the surgery of peripheral nerves. In the last 20 years, we surgically treated 147 patients with radial nerve lesion, which is a remarkable number of cases. The Department for Peripheral Nerve Surgery, Functional Neurosurgery, and Pain Management Surgery at the Clinic for Neurosurgery, Clinical Center of Serbia in Belgrade, Serbia, is a referral center for peripheral nerve injuries and diseases, serving the approximate population of 7 million people of Serbia (28), where every patient in the need for nerve surgery should be automatically referred, as well as the complex patients from the former Yugoslavia region. The present study is the largest in Europe and one of the largest published series on the surgical treatment of radial nerve lesions worldwide (8, 19).

Based on our experience, surgical treatment of radial nerve lesions demands the surgeon to meticulously analyze all aspects of the injury and be aware of the relevant surgical treatment options (17, 29, 30). Detailed insight into etiological and epidemiological characteristics (age, working-age, gender, mechanism, location, extent of an injury, etc.) lead to the clear and more accurate prognosis and recovery expectations. This allows us to achieve best possible outcomes and also to avoid additional surgeries, which may compromise the recovery or even lead to severe consequences.

According to the published literature (26, 31–37), the patients referred for peripheral nerve surgery usually belong to the working population, and majority of them are male. This has also been shown in a study of surgically treated radial nerve lesions in general (38) as well as a study of surgically treated radial nerve lesions associated with humeral shaft fractures (17). The results of our study are in accordance with the results of aforementioned studies.

Regarding some studies (39–49), gender of the patients may be associated with the cause of trauma, which is in line with the results of our study. Most of our patients injured during road traffic accidents, occupational accidents, and fights were males, while most of those who fell were females. These results may be explained by a greater chance for male population to participate in traffic (41–44), fights (48, 49), or work with heavy machines and objects (45, 46), unlike the females, which make them prone to these accidents. On the other hand, the lower body strength of females, in general, may be the reason why they suffer severe injuries in low/energy trauma (50).

Most of the patients in our study were traumatized, and the most common etiology of the nerve lesion was traumatic. These results are in accordance with the results of studies concerning surgically treated radial nerve lesions in general (8, 9), as well as radial nerve lesions associated with humeral shaft fracture (17).

The distribution of mechanisms of traumatic nerve injuries referred for surgery mostly depend on the affected nerve and at what location in the extremity the damage occurred (8, 13, 37). The median and ulnar nerve are more superficial (51), and therefore more exposed to laceration and cutting (13, 37), while the radial nerve, which lays close to the bones, is usually subjected to fracture-related contusion (8, 16). The results of our study may be compared with that of the study by Kim et al. (8). A lower occurrence of our patients with gunshot wounds may be explained by the different firearms available in these two countries (52, 53), as well as the different global peace index (GPI) (54) and different shooting frequency during the study periods (55, 56).

The higher occurrence of iatrogenic nerve lesions in our study, comparing to the study of Kim et al. (8), may be due to different referral of patients in our country. Overall, patients with iatrogenic radial nerve lesions are commonly managed by the surgeons who performed the primary surgery (57). In our country, majority of iatrogenic nerve lesions referred for surgery are managed at our department (14).

Most of the iatrogenic nerve lesions in our study were a consequence of ORIF of the humeral shaft, which is in accordance with the published literature (14, 16, 58). The fact that iatrogenic nerve injuries are a common consequence of the extremity surgery (59) may explain why some of our patients acquired radial nerve lesion during resection of the tumor in the upper arm. The reported cases of iatrogenic radial nerve lesions due to repositioning under general anesthesia are described in the literature (60), and this happened in two of our patients during an emergency surgery. No injection injuries (8) and injuries due to blood pressure cap compression (7) were noted, probably due to the increased awareness of these injuries in the last few decades.

Idiopathic radial nerve lesions may occur due to the nerve entrapment at multiple sites in the upper extremity (7, 61, 62), out of which the most commonly described is at the supinator muscle arch, the arcade of Frohse, which is in accordance with our results. A lower occurrence of patients with radial nerve entrapment in our study, compared to the study of Kim et al., may be due to the low familiarity of treating physicians with this particular entity and a considerable part remaining underdiagnosed.

The most common PNST in the published literature are schwannoma and neurofibroma, usually occurring between the third and fifth decades of life, as solitary lesions, or within neurocutaneous syndromes (15). The schwannomas rarely involve intrafascicular growth of the tumor, unlike neurofibroma, which usually involves several fascicles (63). The results of our study concerning the age, frequency, and presence of infiltration are in accordance with the published literature.

The results of this study suggest that the etiology of radial nerve lesions demanding surgery is most often accidental, rather than health related, and the resources should be directed toward the prevention of such accidents (both traumatic and iatrogenic). Future studies should focus on all aspects of the lesion to better guide the management and potentially predict outcomes of surgical treatment.

The major limitations of this study are its retrospective nature and the involvement of only surgically treated patients. The former may also be the reason for somewhat later referral, as these patients initially seek help from their local medical care providers and get referred only after the definitive failure of conservative treatment. A decent amount of patients received surgical treatment in local centers, especially the iatrogenic cases; therefore, we lack some data that prevent us to analyze the whole patient population.

## Conclusion

The etiology of radial nerve lesion is most often traumatic, and almost all patients belong to the male working-age population. Iatrogenic nerve injuries were frequent and most often a consequence of open reduction and internal fixation of the humeral shaft. The nerve lesions of neoplastic and idiopathic entrapment origin are less frequent in our population.

Based on the fact that traumatic or iatrogenic injuries constitute the majority of cases, with their relevant mechanisms and upper arm predomination, it is crucial to raise awareness and understanding of the radial nerve injuries among orthopedic surgeons to decrease the numbers of these patients and properly preserve or treat them within the initial surgery.

When occurred, the radial nerve lesions may be associated with significant functional and socioeconomic consequences and should be managed by experienced specialists.

## Data Availability

The raw data supporting the conclusions of this article will be made available by the authors, without undue reservation.
